# Multilevel control of run orientation in *Drosophila* larval chemotaxis

**DOI:** 10.3389/fnbeh.2014.00038

**Published:** 2014-02-13

**Authors:** Alex Gomez-Marin, Matthieu Louis

**Affiliations:** ^1^EMBL/CRG Research Unit in Systems Biology, Centre de Regulació Genòmica, Universitat Pombeu FabraBarcelona, Spain; ^2^Champalimaud Neuroscience Programme, Champalimaud Center for the UnknownLisbon, Portugal

**Keywords:** active sensing, chemotaxis, *Drosophila* larva, sensorimotor integration, stereo-olfaction, weathervaning

## Abstract

Chemotaxis is a powerful paradigm to study how orientation behavior is driven by sensory stimulation. *Drosophila* larvae navigate odor gradients by controlling the duration of their runs and the direction of their turns. Straight runs and wide-amplitude turns represent two extremes of a behavioral continuum. Here we establish that, on average, runs curl toward the direction of higher odor concentrations. We find that the orientation and strength of the local odor gradient perpendicular to the direction of motion modulates the orientation of individual runs in a gradual manner. We discuss how this error-correction mechanism, called *weathervaning*, contributes to larval chemotaxis. We use larvae with a genetically modified olfactory system to demonstrate that unilateral function restricted to a single olfactory sensory neuron (OSN) is sufficient to direct weathervaning. Our finding that bilateral sensing is not necessary to control weathervaning highlights the role of temporal sampling. A correlational analysis between sensory inputs and behavioral outputs suggests that weathervaning results from low-amplitude head casts implemented without interruption of the run. In addition, we report the involvement of a sensorimotor memory arising from previous reorientation events. Together, our results indicate that larval chemotaxis combines concurrent orientation strategies that involve complex computations on different timescales.

## Introduction

Along with the nematode *C. elegans*, the *Drosophila* larva offers a genetically tractable system to decipher the neural logic underlying the conversion of dynamic olfactory inputs into directed locomotion (Lockery, 2011; Gomez-Marin and Louis, 2012). Before attempting to crack the neural computation underlying chemotaxis (Olsen and Wilson, 2008), it is necessary to achieve a description of this behavior in terms of elementary motor patterns. The set of basic actions composing larval chemotaxis is reasonably well-known. In the absence of noxious stimulations, larvae display five basic types of movement: forward locomotion (called runs), backward locomotion, stops, turns, and lateral head sweeps (called casts) (Green et al., 1983; Cobb, 1999; Gomez-Marin and Louis, 2012). As the motor patterns underpinning each action are becoming resolved at the level of muscle contraction in single segments of the ventral nerve cord (Lahiri et al., 2011; Heckscher et al., 2012), it is timely to complete our knowledge about the hierarchy of sensorimotor processes that underpin larval chemotaxis.

Larvae are capable of ascending an odor gradient by controlling the duration of their runs and the orientation of their turns (Gomez-Marin et al., 2011; Gershow et al., 2012). Turns are facilitated during movements down the gradient whereas they are suppressed during movements up the gradient. On average, turns are oriented toward the direction of the local odor gradient. This directional bias results from an active sampling process where the local gradient is detected through lateral head casts prior to the implementation of a turn (Gomez-Marin et al., 2011; Gershow et al., 2012). A similar algorithm applies to chemotaxis in *C. elegans*, except for the difference that the turns are not directed toward the local gradient. Turning maneuvers, called pirouettes, induce 180° changes of orientation without fine directional correction with respect to odor gradient. While runs were initially approximated as straight segments in *C. elegans* (Pierce-Shimomura et al., 1999), it was later discovered that runs bend toward the direction of higher concentrations—a process called *weathervaning* or *klinotaxis* (Iino and Yoshida, 2009; Izquierdo and Lockery, 2010; Lockery, 2011). In the absence of weathervaning, reorientation through the pirouette mechanism is insufficient to account for the navigational performances in odor gradients (Izquierdo and Lockery, 2010). Weathervaning appears to be mediated by “shallow turns” during which changes in orientation are reduced compared to those of pirouettes (Kim et al., 2011).

Here, we test the relevance of weathervaning in the *Drosophila* larva. Like for *C. elegans*, we find that larvae are capable of biasing the direction of individual runs toward the local odor gradient. The strength and sign of the modulation are proportional to the lateral component of the sensory gradient, when projected onto the animal's orientation. Engineering larvae with genetically modified olfactory systems with a single functional olfactory sensory neuron (OSN), we rule out the possibility that run biases rely on the detection of concentration differences between the left and right olfactory organs of the larva (stereo-olfaction). We speculate that weathervaning relies on the detection of temporal changes in the stimulus intensity, rather than on instantaneous bilateral comparisons. From a careful inspection of the sensorimotor trajectories of larvae, we propose that runs are directed toward the direction of higher concentrations based on the detection of minute changes in stimulus intensity during low-amplitude head casts. According to this model, head casts would contribute to the orientation behavior of larvae in two significant ways. First, it would mediate the detection of local concentration changes along the axis perpendicular to the direction of motion. This would induce smooth reorientation corrections as the animal crawls forward. Second, it would permit larvae to pinpoint the direction of higher concentrations through large head casts during the preparation of a turn. We also find a subtle modulation of run dynamics by the projection of the gradient parallel to the direction of motion, and a contribution of a short-term sensorimotor memory to the navigation behavior. Overall, these findings refine our knowledge about the multilevel hierarchy of sensorimotor processes that control larval chemotaxis.

## Materials and methods

### Fly genetics

Fly stocks were kept in a 12 h dark–light cycle and maintained on conventional cornmeal-agar molasses medium at 22°C. w1118 was used as a “wild-type” control. We genetically engineered larvae with one class of functional OSNs—one OSN on each side of the head—by restoring the expression of *Orco* (Vosshall and Hansson, 2011) in the *Or42b*-expressing ORNs. Ectopic expression of *Or42b* was induced in all larval OSNs by driving the expression of UAS-*Or42b* with the *Orco*-Gal4 driver (Larsson et al., 2004). The UAS-*Or42b* transgene was obtained by cloning full-length *Or42b* complementary DNA into a pUAST vector and by generating transformants according to standard methods. As described elsewhere (Louis et al., 2008), unilateral *Or42b*-functional larvae were obtained by using an *Orco* UAS-flipout construct to stochastically restore the expression of *Orco* in the *Or42b*-expressing OSN by crossing the parental lines *w*; *Or42b*-Gal4; hs-Flp, *Orco*^2^ with *w*; UAS->RFP-stop>GFP-Orco; *Orco*^1^ where > denotes FRT sites. The *Or42b*-Gal4 driver used in the present work was published in a previous study (Fishilevich et al., 2005). Larvae were raised at 28.5°C and subjected to a 15-min heat shock at 36°C 48 h after egg laying. Individuals were not pre-sorted by phenotypes before the behavioral experiments. After each experiment, the anterior tip of the larva was isolated, fixed in 4% formaldehyde solution and rinsed abundantly with PBS. The phenotype of each larva was assessed by confocal microscopy based on the detection of GFP expression.

### Odor gradient reconstruction

Experiments were conducted with ethyl butyrate (Sigma-Aldrich) at the highest level of purity. The odor was diluted in paraffin oil. As described in Gomez-Marin et al. (2011), odor gradients were created in arenas composed of a stack of 96-well plate lids. The odor source consisted in 10 μL droplet at a concentration of 30 mM loaded in one of the wells at the center of the top lid. The odor gradient in gaseous phases was reconstructed and interpolated according to the procedure described in Louis et al. (2008), Gomez-Marin et al. (2011).

### Behavioral arena and preparation of animals

All behavioral experiments involved third instar foraging larvae tested during day time. Room temperature was kept between 22 and 24°C, and relative humidity fluctuated between 50 and 60%. A light pad (Slimlite Lightbox, Kaiser) illuminated the arena from above creating uniform daylight conditions. Larvae were washed with a solution of 15% sucrose. Individuals were tested within 90 min after introduction of sucrose into the food vial. The chemotaxis assay consisted of a 3% agarose slab coating the top surface of a rectangular plate lid. Immediately after the introduction of a larva in the arena, the top lid was inverted onto the agarose layer. The starting position of the animal and the location of the odor droplet coincided with the center of arena. Lids and agarose layers were not reused across experiments. Behavioral tracking lasted a maximum of 5 min or was interrupted if the larva touched the plate walls.

### Tracking and image processing

The locomotor behavior of individual larvae was tracked and analyzed by the freely-available custom-made software *SOS-track* described in Gomez-Marin et al. (2012). Recording of the larval body postures was carried out using a video camera (Stingray Camera, Allied Vision; Computer lens, 12–36 mm, 1:2:8, 2/3″ C) at a spatial resolution of 90 μm per pixel placed underneath the assay plates. Frames were acquired at 7 Hz and preprocessed online. In an offline routine, the skeleton of the animal was computed and its endpoints were automatically classified as head or tail based on a proximity rule. Raw trajectories of the location of the animal's head, tail and centroid were smoothed by a third-order polynomial fit using a 2-s time window.

### Quantification of run reorientation and local stimulus gradients

To quantify the correlation between the instantaneous reorientation bias of single runs and the local stimulus gradient, we defined (1) a measure of the curvature of run segments and (2) an estimate of the direction and strength of the local gradient relative to the animal. The direction of motion of the larva's body is described by the body angle, defined as the angle between the body axis and the long axis of the arena. This measure does not rely on an interpolation of the centroid trajectory and so it remains well-defined even in the absence of spatial translation of the body. The body angle is a robust measure of the animal's absolute orientation and its time derivative quantifies the instantaneous reorientation rate (ϕ). Traditionally, the path curvature is considered as a static geometrical measure with units of degrees per millimeter, see for instance (Iino and Yoshida, 2009). Alternatively, the directional bias of a run as the animal moves forward can be accessed directly through the reorientation speed measured in degrees per second. Both ways of assessing the local curvature require discretizing the trajectory in sampling intervals (Kafkafi and Elmer, 2005). We used a sampling interval corresponding to the tracking frequency (7 Hz). The latter approach—kinematic rather than geometric—has the advantage that it evolves in time like the rest of sensorimotor variables considered during larval chemotaxis. It is decoupled from translation and, as explained above, it is well-defined irrespectively of the speed of the animal (even during pauses in forward locomotion). We therefore chose to quantify the local curvature of runs through the body reorientation dynamics. Nevertheless, our main results hold for other definitions of the local curvature (Supplementary Figure 1).

In our analysis, turns were excluded from trajectory segments. As discussed in Gomez-Marin et al. (2011), the crossover observed in the distribution of instantaneous reorientation rates allows us to identify turns, which are not the focus of the present analysis. Accordingly, we excluded segments comprising any instantaneous absolute reorientation speed >12° per second. To avoid including into our data trajectory segments data that may contain the immediate influence of recent turning events—either reorientation right after the execution of turns or its preparation—we excluded a time window within runs in our analyses. We explored the effect of the window size on the sensory modulation of the reorientation and found 1 s as the optimal threshold (Supplementary Figure 2D). Nevertheless, the phenomenon is still clearly present for several exclusion windows before and after every turn event, thereby showing that our main results are not contingent on such a filtering.

To estimate the effects of the stimulus gradient when projected on the body axis of the animal, we calculated the bearing angle (β) between the larva's heading and the local direction of maximum concentration increase (Gomez-Marin et al., 2011). Using the SOS-track software, we mapped the position of the animal's head and centroid onto the reconstructed odor gradient, obtaining quantitative estimates of the corresponding odor dynamics experienced by the larva during its motion in the arena. We calculate the local odor gradient that is then decomposed into its lateral and parallel projections with respect to the animal body axis (equivalent to the direction of motion). With these metrics, we can study how reorientation is modulated by the local gradient and by the bearing angle, which offers a finer animal-centric descriptor than the heading to the odor source position. Using the weathervane analogy, when the local gradient is leftward from the animal's perspective, we expect the larva to rotate to the left, thus improving its local alignment with the gradient. Finally, the time courses of the odor concentration at the tip of the head of the larva is used as an approximation of the sensory dynamics experienced by the larva during chemotaxis.

### Behavioral statistical analysis

Our behavioral analyses aim to establish a quantitative and statistical relationship between the instantaneous reorientation rate and the lateral projection of the local odor gradient. We compute this relationship through (1) the mean reorientation as a function of the gradient strength and (2) the distribution of reorientations for positive and negative gradients (where positive refers to left side by default). Throughout the study we pooled run segments across animals. This pooling is necessary to build the reorientation distributions, due to the relatively small number of run events that are associated with each animal (approximately 30). Nevertheless, we plot the mean instantaneous reorientation rate for every animal binning the lateral sensory gradient into positive (leftwards) and negative (rightwards) values (Supplementary Figure 3). Upon averaging across larvae, we find the same trend as after pooling all runs.

For genotypes with an altered olfactory system, statistical differences in the distributions of reorientation conditioned to positive and negative lateral gradients were assessed by following two complementary approaches. First, we addressed whether a side bias exists by comparing the means to zero using standard *t*-test statistics. Second, to rank the intensity of the effect, we calculated the distance between the positive and negative distributions for every experimental group by means of the Kullback-Leibler distance (or relative entropy), which can be defined as the sum over *i* of *P*(*i*)^*^ln(*P*(*i*)/*Q*(*i*)), where *P*(*i*) and *Q*(*i*) are two probability distributions defined over the same domain discretized in *N* bins. The relative entropy is an information theory metrics that weights the logarithm of the ratio of both distributions. This allows taking differences observed across the whole probability distribution into account. The entropy is a convenient metric that yields a non-negative scalar value to rank the asymmetries between the distributions of different genotypes. To generate adequate statistics to compare different groups, we bootstrapped with replacement the left and right gradient reorientation distributions (Efron and Tibshirani, 1993), creating 1000 replicas of each class, from which we calculated the relative entropies to then apply a two-sample *t*-test for difference in their mean.

## Results

### Weathervaning in larval chemotaxis: sensory modulation of run orientation

**Figure 1:**
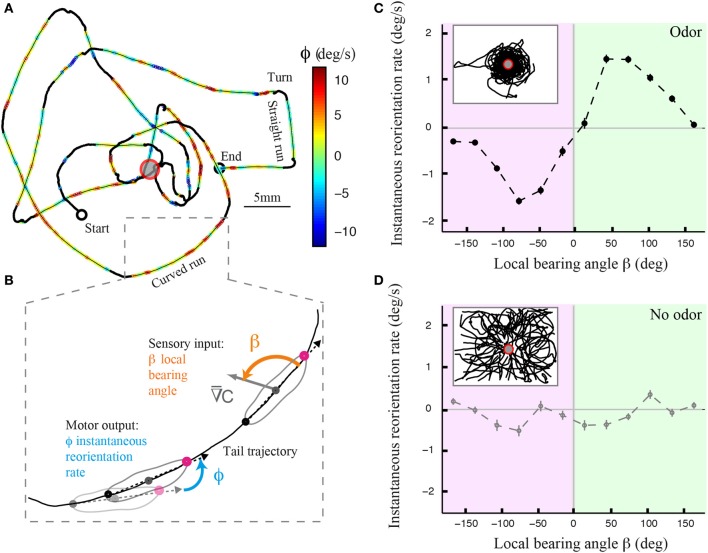
**Directional modulation of runs in *Drosophila* larval chemotaxis. (A)** Illustrative trajectory of a larva navigating near an odor source (red ring). The trajectory can be decomposed into turns (black), straight runs, and curved runs. Runs are color-coded based on the larva's reorientation rate or instantaneous change in orientation (see Materials and Methods). Yellow-to-red color range denotes counterclockwise rotations as the animal progresses forward. Green-to-blue denotes clockwise rotations. **(B)** Close-up view of a curved run trajectory (tail position) where the instantaneous reorientation rate (ϕ) and local bearing angle to the stimulus (β) are visually illustrated. The relationship between these two variables allows us to determine the contribution of weathervaning to the reorientation process. **(C)** Dependency of the averaged reorientation rate on the local bearing angle for wild type larvae tested in the presence of a sensory gradient created by an odor droplet of 30 mM ethyl butyrate. Inset shows a superposition of 42 trajectories of wild-type larvae. The reorientation bias is maximum when the sensory gradient is lateral to the animal and it is correlated with the direction of the stimulus. The bias decays to zero when the gradient is parallel to the animal, either facing up the gradient or down the gradient. The bearing angle values are represented in 12 bins of 30°. **(D)** Same plot as in **(C)** for wild type larvae (*N* = 17) tested in the absence of odor, where the fictive local bearing angle is computed based on the odor landscape used in **(C)**. The absence of directional modulation establishes that the correlation between reorientation rate and local bearing is due to the odor gradient and that it does not result from the geometry of the arena. In **(C,D)**, the average reorientation rates are calculated from bins including between *N*_min_ = 1096 and *N*_max_ = 6448 data points.

Using an assay and tracking software described elsewhere (Gomez-Marin et al., 2011; Gomez-Marin and Louis, 2012), we monitored the behavior of individual larvae in controlled gradients of ethyl butyrate. Figure 1A illustrates the trajectory of a wild type larva after its decomposition into runs and turning maneuvers that include head casts. A qualitative inspection of the trajectory segments reveals that runs are not perfectly straight but that they are curved (Figure 1B). To define whether the curvature of the runs is correlated with the local odor gradient, we introduced the instantaneous reorientation rate ϕ (Figure 1B). The curvature of the trajectory is modulated by the direction of the local odor gradient, quantified by the bearing angle β (Figure 1C). This correlation is inexistent when the larva evolves in an arena devoid of odor (Figure 1D), ruling out effects purely related to the geometry of the assay and underscoring the sensory nature of the phenomenon.

**Figure 2:**
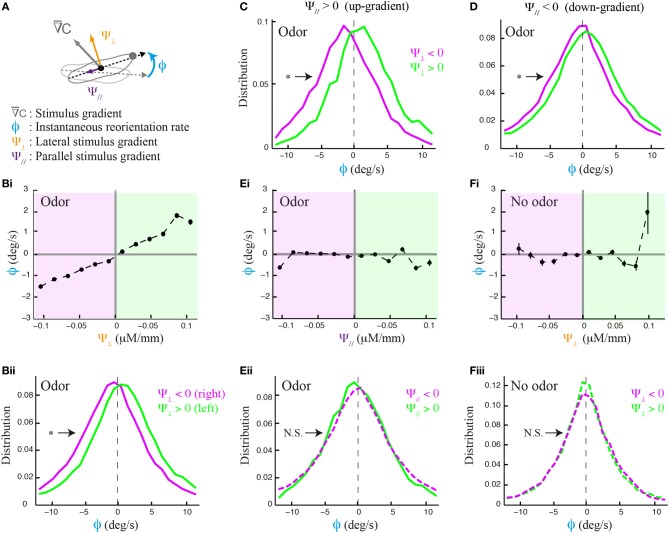
**Effects of lateral and translational stimulus gradients on the weathervane corrections of wild type larvae. (A)** Illustration of the three main variables applied to characterize weathervaning: reorientation rate (ϕ) and projection of the local odor gradient on the axis tangent to the direction of motion (translational gradient, Ψ_//_) and projection perpendicular to the direction of motion (lateral gradient, Ψ_⊥_). All panels correspond to the behavior of wild type larvae. **(Bi)** Lateral gradient modulates run directionality both in sign and in strength (linear correlation: slope = 15.5, *R*^2^ = 0.975). Gradient values are represented in 12 bins of 0.02 μM/mm. **(Bii)** Distributions of reorientation rates during runs are shifted to the left or the right according to the direction of the lateral gradient. By convention, positive values point to the left (green) and negative values point to the right (magenta). Plain lines indicate statistically significant differences between 0 and the mean of the distribution (one-sample *t*-test, *p* < 0.001). The star indicates a significant difference between the means of the left and right distributions (two-sample *t*-test, *p* < 0.001). **(C,D)** Distributions of reorientation rates restricted to runs oriented up-gradient and down-gradient (positive and negative translational gradient, respectively). A stronger modulation of the reorientation rate through the lateral gradient is found for up-gradient runs. Same statistical representation as in **(Bii)**. **(Ei)** In presence of odor, the local odor gradient parallel to the animal does not condition run directionality (slope of linear regression equal to −0.86, *R*^2^ = 0.043). **(Eii)** Reorientation rate distributions restricted to left and right lateral gradients are not significantly different from each other (two-sample *t*-test, *p* = 0.06), nor is the mean of the distribution different from 0 when the gradient points to the right (one-sample *t*-test, *p* = 0.2), although a statistically significant difference is found for the mean when the gradient points to the left (one-sample *t*-test, *p* = 0.001). **(Fi)** Control condition in absence of odor shows no modulation of the lateral gradient (slope of linear regression equal to 3.0, *R*^2^ = 0.089). **(Fii)** Reorientation distributions are not significantly different from zero (one-sample *t*-test, *p* > 0.12), neither is the difference between their means (two-sample *t*-test, *p* > 0.8) indicating that the asymmetric modulation of individual runs is caused by the sensory gradient. In **(Bi,Ei,Fi)**, the average reorientation rates are calculated from bins including between *N*_min_ = 247 and *N*_max_ = 7490 data points.

The local odor gradient is a vector that can be decomposed into two components: its projection tangent to axis of motion and that orthogonal to it (Figure 2A). We refer to the former as the translational gradient (Ψ_//_) and to the later as the lateral gradient (Ψ_⊥_). We tested whether larvae can estimate the strength and sign of the lateral gradient during forward motion (Figure 2B) and found that run reorientation scales with the lateral gradient (Figure 2Bi). As shown in Supplementary Figure 3, this trend holds when the analysis is restricted to single larvae. By clustering runs oriented up-gradient (Ψ_//_ > 0, Figure 2C) and down-gradient (Ψ_//_ < 0, Figure 2D), we conclude that the strength of the directional bias is considerably larger when larvae move toward the source. Next, we asked whether the translational gradient has a direct effect on weathervaning (Figure 2E), but found no significant bias. As a control, we found that the trend in Figure 2B vanishes in absence of odor (Figure 2F) and also in the anosmic *Orco* null background in presence of odor (Supplementary Figure 4). These results indicate that weathervaning is controlled by the olfactory inputs. Since the bearing (β, Figure 1B) captures only the direction of the gradient and not its strength, we adopt a representation based on the lateral gradient in the rest of the work (Ψ_⊥_, Figure 2A).

Figures 1, 2 show that the weathervane corrections cannot bias run directionality toward the gradient when the larva's body axis is totally parallel to it (oriented either up-gradient or down-gradient). Yet the amplitude of the weathervane corrections is slightly larger when the larva has the sources at its back rather than in front of it (Supplementary Figure 5A). As will be further addressed below, this difference between up-gradient and down-gradient orientations establishes that the direction of the translational gradient can also modulate run dynamics during chemotaxis. Overall, the side of the reorientation bias is primarily determined by the direction of the lateral gradient. Weathervaning in the *Drosophila* larva constitutes a continuous orientation mechanism that relies on the detection of the lateral gradient to induce smooth correction in the direction of motion toward the odor source.

### Nature of the orientation mechanism underlying run reorientations

While the translational gradient can be inferred through the detection of dynamic changes in odor concentration during forward locomotion, the detection of asymmetries in the lateral gradient poses a conceptual challenge: temporal changes in concentration can result from the detection of the lateral gradient, the translational gradients, or a combination of both. The larval olfactory system appears to be adequately designed to tell apart the two dimensions of the gradient. Unlike the adult fly (Stocker et al., 1990; Gaudry et al., 2013), the projection of the left and right OSNs is purely ipsilateral (Gomez-Marin et al., 2010). This anatomical compartmentalization could enable the larva to detect the lateral gradient through instantaneous comparisons between the left and the right olfactory inputs. Although such a mechanism is not necessary to explain the orientation of turns (Louis et al., 2008; Gomez-Marin et al., 2011), we tested the relevance of spatial comparisons to the control of weathervaning during runs.

In past works, we devised a genetic strategy to create larvae with unilateral olfactory function restricted to a single OSN, that expressing the *Or42a* odor receptor (Louis et al., 2008). We observed that individuals with a single functional *Or42a*-expressing OSN show repulsion to high concentrations of ethyl butyrate—the odor tested here—whereas wild type demonstrates strong attraction to the odor (Asahina et al., 2009; Gomez-Marin et al., 2011). To unambiguously focus the present analysis on attractive behavior and to avoid confounding situations where repulsion could mask weathervaning, we searched for another OSN that elicits robust attraction to ethyl butyrate without obvious signs of aversion. Ethyl butyrate induces significant activity in only three types of OSNs: *Or35a*, *Or42a*, and *Or42b* (Asahina et al., 2009). As larvae with an *Or35a*-functional OSN are unable to chemotax, we focused on *Or42b* and adapted our stochastic rescue strategy to restrict olfactory function to the left or to right side (Materials and Methods). We then tested larvae with unilateral and bilateral functions in the same odor gradients as wild type.

**Figure 3:**
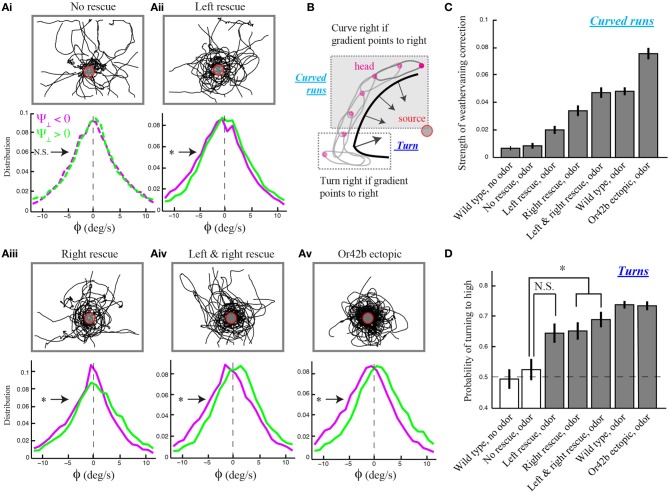
**Temporal nature of the weathervane correction in larvae with genetically modified olfactory systems. (A)** Overlaid trajectories (top) and reorientation rate distributions restricted to left and right lateral gradients (bottom) for larvae with different numbers of functional olfactory sensory neurons (OSNs) expressing the *Or42b* odorant receptor gene: no rescue (anosmic, *N* = 24 animals), unilateral left rescue (*N* = 21), unilateral right rescue (*N* = 20), bilateral rescue (*N* = 23), and ectopic expression of *Or42b* in all 21 OSNs (*N* = 33). Plain lines indicate statistically significant differences of the distribution means with 0 (one-sample *t*-test, *p* < 0.01). The star indicates a significant difference between the means of the left and right distributions (two-sample *t*-test, *p* < 0.001). All manipulations, except for the no-rescue condition (*t*-test, *p* > 0.05), show statistically significant differences in the modulation of the distributions to the direction of the lateral gradient, ruling out that bilateral comparisons are necessary for weathervane corrections in response to olfactory stimuli. **(B)** Schematic drawing illustrating the two main types of reorientation events: turns (large changes in orientation implemented through high-amplitude head casts preceded by a brief pause in locomotion) and weathervaning (modest changes in orientation during curved runs). For larval chemotaxis, turns were introduced in Gomez-Marin et al. (2011) while weathervaning is examined in the present study. **(C)** Quantification of weathervane corrections. Genotypes introduced in **(A)** together with wild type larvae tested in the presence and the absence of odor. Having shown the existence of weathervaning for larvae with at least one functional OSN **(A)**, we estimate the strength of weathervaning through the relative entropy between the reorientation distributions restricted to the left and the right lateral gradients (**A** and Materials and Methods). The distance between left and right distributions increases with the number of functional OSNs. A bootstrapping method with replacement (1000 replicas) was applied to assess the statistical significance of difference between entropy estimates. Error bars represent SD. This procedure supports that right rescues undergo stronger corrections than left rescues, and that corrections of bilateral rescues are stronger than those of unilateral rescues (two-sample *t*-test, *p* < 0.001). **(D)** Quantification of orientation corrections through turns. Fraction of turns directed toward the higher odor concentrations. All genotypes with at least one functional OSN tested in the presence of odor show a significant turning bias to high when compared to the no-rescue condition (One-Way ANOVA followed by a *post-hoc* analysis with Bonferroni correction for multiple comparisons, *p* < 0.05). As indicated by the star, a significant difference is found between the no-rescue and larvae with unilateral right and bilateral functions (^*^p < 0.005). Error bars denote s.e.m.

We found that *Or42b*-OSN single-functional larvae with unilateral function exhibit the reorientation bias observed in wild type, while the strength of the weathervane effect is reduced compared to larvae with bilateral function (Figure 3A). This result was established in three independent manners. First, we checked for a non-zero bias in the mean of the reorientation distributions upon lateral gradient with positive (leftwards) and negative (rightwards) sign. Second, we quantified the magnitude of the weathervane effect by comparing both distributions directly through their relative entropy (Materials and Methods). Third, we looked at the linear correlations between the reorientation rate and the lateral gradient for all genotypes (Supplementary Figure 6). We found that stereo-olfaction is not necessary for larvae to orient their runs through weathervaning. Moreover, the relative entropy indicates that larvae with unilateral function on the right side detect better the sensory asymmetries in the spatial gradient than larvae with unilateral function on the left side (Figures 3Aii,iii). This result expands previous observations supporting the existence of handedness in the larval olfactory circuit (Louis et al., 2008).

As individuals with bilateral function perform better than individuals with unilateral function, weathervaning benefits from an enhancement in the detection of temporal changes in the olfactory inputs (Figures 3Aii–iv). To test this reasoning further, we engineered larvae with an olfactory system exquisitely tuned to ethyl butyrate by ectopically expressing the *Or42b* receptor in all 21 OSNs (Materials and Methods). Larvae with ectopic expression of *Or42b* display a level of weathervane correction stronger than individuals with bilateral olfactory function and wild type larvae (Figures 3A–C). Based on the entropy metric, we can rank the accuracy of the weathervane correction achieved with different numbers of functional OSNs tuned to the test odor: no rescue, unilateral functional left, unilateral functional right, bilateral functional, wild type and ectopic expression of *Or42b* in all OSNs (Figure 3C). The correlation between the reorientation performance and the number of OSNs sensitive to the odor supports the temporal sampling hypothesis rather than instantaneous bilateral comparisons as the control mechanism underlying the weathervaning behavior.

### Interplay between curved runs and turns through motor memories

**Figure 4:**
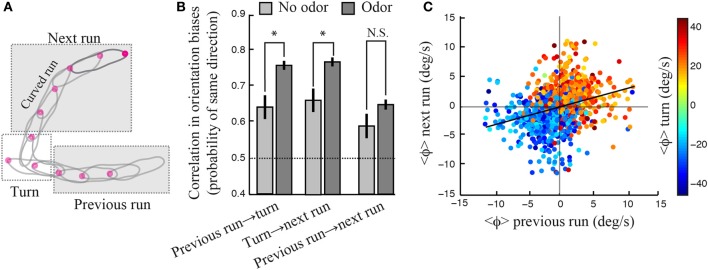
**Contribution of motor memory to reorientation maneuvers. (A)** Schematic drawing illustrating a run leading to a turn followed by a run. **(B)** Conditional probability that the directional bias between two consecutive events—run or turn—be the same. In the absence of odor (light gray bar), wild type larvae show a significant correlation between the direction of consecutive runs and turns, which demonstrates the existence of a purely motor memory. This directional bias is stronger upon odor stimulation, suggesting that sensory stimulation results in a behavioral enhancement. There is a small, yet significant, correlation between the directions of consecutive runs considered irrespectively to the turn in-between. However, this correlation is not strengthened in the presence of odor (two-sample *t*-test, *p* > 0.09). Standard *t*-tests were used to compare the means observed in the absence and the presence of odor (^*^p < 0.001). The difference of the mean probability with chance (0.5) is highly significant for every group (one-sample *t*-test, *p* < 0.001), except for the no-odor previous-run-to-next-turn condition (*p* = 0.009). Error bars denote s.e.m. **(C)** Mean reorientation rate of a given run and the subsequent run (*N* = 1236). The color code indicates the mean reorientation rate during the turns linking two runs. Although the linear correlation is weak (slope = 0.31, *R*^2^ = 0.0969), the grouping of points by color indicates that weathervaning is contingent on the directionality of past runs and turns. In **(B,C)**, the reorientation bias associated with a given run is computed as the average of the instantaneous reorientation rates occurring during this run.

Larval locomotion displays curved runs and turns preceded by high-amplitude head casts (Figure 3B). To explore the existence of a relationship between the directional bias of curved runs and turns, we quantified the reorientation performances associated with individual turns by calculating the percentage of turns oriented toward the direction of the local odor gradient (Gomez-Marin et al., 2011). Using this metric, we observed that larvae with unilateral function restricted to the right side show a significantly larger fraction of turns toward the gradient than the no-rescue control. The same holds for larvae with bilateral olfactory function. A substantial, yet non-significant, turn bias is found for individuals with unilateral left function (Figure 3D). While the ability of unilateral right *Or42b* rescues (larvae with a single OSN functional on the right side) to direct their turns is a novel finding, our results are in line with our previous observation that reorientation behavior does not require stereo-olfaction with a single functional OSN expressing the Or42a odorant receptor (Louis et al., 2008). We find that the percentage of “correct” turns toward higher concentrations tend to increase with the number of OSNs sensitive to ethyl butyrate. We hypothesize that this behavioral improvement can be due to (1) an increase in the signal detected through high-amplitude head casts that precede a turn and/or (2) an improvement in the directional information accumulated during runs. The first hypothesis implies that the encoding of concentration changes during head casts benefits from a larger number of olfactory receptors. The second hypothesis suggests the existence of a correlation between the weathervane bias of consecutive runs and the direction of the flanking turns (Figure 4).

Considering the run that precedes and follows a given turn (Figure 4A), we find that the average direction of a turn is biased toward the direction of weathervane corrections of the previous run (Figure 4B, left condition), which are simply calculated by averaging the reorientation rate for every individual run segment. Similarly, the average direction of a turn is correlated with the weathervane bias of the following run (Figure 4B, center condition). Interestingly, a significant correlation between the orientation of runs and turns is observed even in the absence of odor (light gray bars, Figure 4B). This reveals the existence of a motor memory coupling subsequent run and turn decisions. In addition we find that the correlation between the directions of consecutive runs and turns increases significantly upon odor stimulation. Together, these results indicate the existence of an intricate interplay in the directional control of consecutive runs and turns. Such a relationship is weaker, but still present, when the correlation between consecutive runs is examined irrespectively to the turn in-between (Figure 4B, right condition and Figure 4C). The information acquired about the direction of the local odor gradient during a run appears to contribute to the directional bias of the next turn. Similarly, the direction of a turn influences the directional bias of the next run.

### Effect of the translational gradient on run dynamics

**Figure 5:**
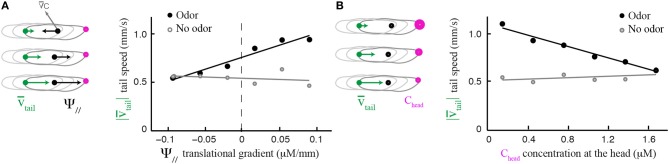
**Modulation of run dynamics by the translational gradient. (A)** Positive correlation between mean tail speed and the strength of the parallel odor gradient (6 bins). In presence of odor, wild type larvae increase their run speed proportionally to the strength of the gradient (slope = 2.46, *R*^2^ = 0.94) whereas locomotion is unaffected in the absence of odor (slope = −0.25, *R*^2^ = 0.08). **(B)** Negative correlation between mean tail speed and stimulus concentration experienced at the head (6 bins). The speed of forward locomotion is inversely proportional to the absolute concentration of the odor (slope = −0.29, *R*^2^ = 0.97). When stimulated by the odor, larvae tend to accelerate as the odor concentration decreases. In the absence of odor, the correlation is very weak (slope = 0.04, *R*^2^ = 0.24). In the analyses shown in **(A,B)**, the initial and final 3 s of each run were excluded to avoid the inclusion of slowing-down and speeding-up effects preceding or following turns.

Although the translational gradient does not induce a weathervane bias *per se*, its direction (up-gradient or down-gradient) influences the amplitude of the weathervane correction (Figures 2C,D). We explore the sensorimotor kinematics of the larva and asked whether the translational gradient bias can modulate other aspects of the run dynamics. Figure 5A establishes that the speed of translation of the tail—the most robust locus of the larva's body to assess forward locomotion independently of body bending—is correlated with the strength of the translational gradient. In presence of odor, larvae tend to accelerate when they move up-gradient; they decelerate when they move down-gradient (black circles). This relationship is preserved when the first and last 3 s of each run are excluded, ruling out artifacts due to the slowing of locomotion in preparation of turn (Supplementary Figure 2). When larvae evolve in an arena devoid of odor, the correlation between run speed and a fictive translational gradient is absent (Figures 5A,B, light gray circles). Since the modulation in run speed is modest (one third around the mean speed), its contribution to the overall reorientation algorithm might be secondary compared to the correction in orientation resulting from weathervaning and turns upon the integration of the translational and lateral gradients (Gomez-Marin et al., 2011; Gomez-Marin and Louis, 2012). However, these findings suggest that a combination of multilevel orientation strategies participate in the control of larval chemotaxis.

### Weathervane corrections improve reorientation and contribute to chemotaxis

**Figure 6:**
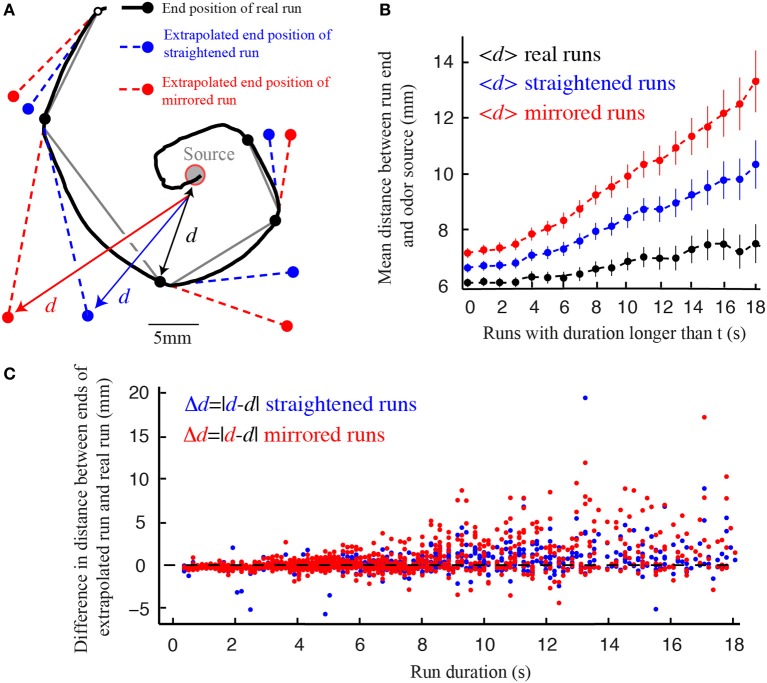
**Weathervane corrections improve reorientation. (A)** Real trajectory of a wild type larva for which the experimentally observed end position of each run (black) is compared with the extrapolated end position of virtual runs: (1) fictive run whose path is straight in the direction tangent to the beginning of the real run (blue) and (2) mirrored run curling in the opposite direction of the real run (red). The distances (*d*) from the odor source to the end point of each run are calculated. **(B)** Average distances between the source and the end position of the real, straight and mirrored runs, as a function of the cumulative run duration (*t*). On average, real runs bring the larva closer to the source for any run duration. Straight runs lead to better performances than mirrored runs. Error bars represent s.e.m. **(C)** Source-to-endpoint distance differences between the real and virtual runs, both for straight runs (blue dots) and mirrored runs (red dots). Individual events are ranked by duration (*N* = 1236 runs for wild type larvae in the presence of odor). Individual virtual run events rarely do better than real runs subjected to weathervaning.

In analogy with the same process in *C. elegans*, we call the smooth reorientation of runs *weathervaning* (Iino and Yoshida, 2009; Kim et al., 2011). This continuous correction has been shown to significantly improve the chemotactic performances of *C. elegans* (Iino and Yoshida, 2009; Lockery, 2011). Similarly, we find that weathervaning corrections bring the larva closer to the odor source at the end of a run. To this end, we compare the difference in distance separating real runs and extrapolated runs that would not be affected by weathervaning (straight virtual runs) or that would curve on the side opposite to the real run (mirrored virtual runs) (Figure 6). The improvement achieved through weathervaning is obvious. In addition, we show that the amplitude of the weathervane correction is a function of the strength of the lateral gradient (Figure 2). When the larva moves up-gradient, the amplitude of the weathervane correction is stronger than when the larva moves down-gradient (Figures 2C,D and Supplementary Figure 5). Hence, weathervaning constitutes an error-correction mechanism that orients individual runs toward the odor source.

## Discussion

To date, runs separating turns were approximated as straight segments in the analyses of sensory navigation in the *Drosophila* larva. We have completed this model by taking into account curved runs and by demonstrating the existence of a side bias of individual runs toward the direction of the local odor gradient. We find that the amplitude of the curvature of the trajectory is a function of the strength of the gradient parallel to the direction of motion, suggesting that weathervaning is regulated in a continuous manner through active sensing. To dissociate whether the underlying computation of this newly described chemotactic strategy is based on bilateral sensing or purely on temporal computation, we engineered larvae with unilateral olfactory function. Surprisingly, we discovered that larvae with a peripheral sensory system restricted to just a single functional neuron are able to control when to terminate a run as well as where to reorient through the smooth corrections induced by weathervaning and wide-amplitude turns.

**Figure 7:**
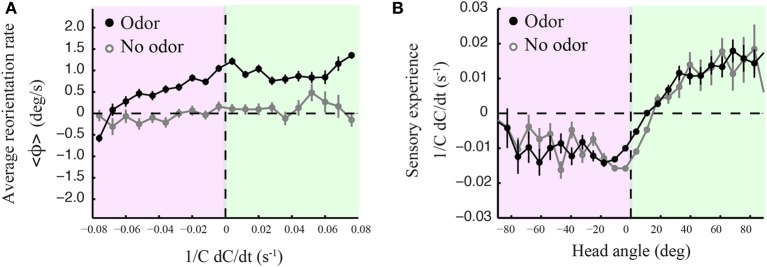
**Curved runs, head casts and olfactory experience. (A)** Averaged instantaneous reorientation rate (ϕ) as a function of the sensory experience measured at the head (*1/C dC/dt*). Here positive and negative signs of reorientation rates imply reorienting toward and away from the gradient, respectively. The black line corresponds to wild type larvae tested in presence of odor. The light gray line corresponds to the no-odor control carried out with wild type. In the presence of odor, the signed average reorientation rate is positive nearly throughout the range of sensory experience, whether the instantaneous stimulus concentration derivative at the head is positive (green quadrant) or negative (red quadrant). **(B)** Averaged sensory experience as a function of the signed head angle where positive angles point toward the local gradient, as for the reorientation rate of **(A)**. Negative sensory experiences are obtained when the head points away from the gradient. However, straight postures and small positive head angles correspond to negative sensory experiences (both in the presence and absence of odor), suggesting that motion tends to bring the larva away from the position of the odor source, which leads to an average decrease in odor concentration. In **(A,B)**, error bars represent s.e.m. The distributions arise from *N* = 1236 runs for the odor condition and *N* = 210 runs for the no-odor control. In **(A)**, averages are calculated from bins of 0.008 s^−1^ including between *N*_min_= 273 and *N*_max_ = 3519 data points. In **(B)**, averages are calculated from bins of 7.2° including between *N*_min_ = 106 and *N*_max_ = 11169 data points.

**Figure 8:**
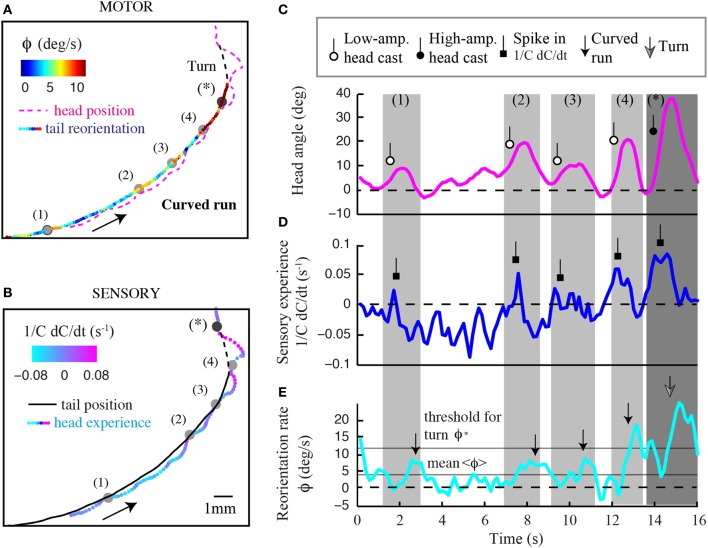
**Contribution of head sampling to weathervaning and wide-amplitude turns.** Illustration of sensorimotor degrees of freedom in a representative run undergoing weathervane corrections. **(A)** Motor dimension of the run displaying the head position (dashed magenta line) and tail position color-coded with the instantaneous reorientation rate. **(B)** Sensory dimension of the run displaying the tail position (plain black line) and head trajectory color-coded with temporal changes of odor concentration (*1/C dC/dt*). Numbered events represent low-amplitude head casts directing weathervaning. **(C)** Time course of the larval head angle dynamics corresponding to the trajectory inset shown in **(A,B)**. Low-amplitude head casts are marked by a lariat symbol to compare event sequence across sensorimotor variables. **(D)** Temporal changes in sensory input at the animal's head. Increase in sensory experience due to low-amplitude head casts are marked by a square symbol. **(E)** Time course of the instantaneous reorientation rate (ϕ). Weathervaning corrections (small arrow) tend to follow peaks in sensory experience, which result from low-amplitude head casts during forward locomotion. About 13 s after the run onset, the crossing of the threshold for turn (ϕ^*^) does not lead to a turn as the reorientation rate returns below the threshold within less than 1 s (Gomez-Marin et al., 2011). A fully developed turn takes place where indicated by the large arrow. The sequence in **(C–E)** suggests that head casts are involved in the stimulus sampling before implementing both weathervaning and turns.

The precise mechanistic interpretation of these findings is limited by the tools of our approach; despite the use of high-resolution and quantitative behavioral analyses, we are not in a position to decouple the sensorimotor loop in freely moving larvae, as has been initiated for *C. elegans* by combining modeling and *in vivo* calcium imaging (Izquierdo and Lockery, 2010; Larsch et al., 2013). This would allow designing, at will, particular sensory dynamics upon certain motor actions and test the validity of causality relationships. We nonetheless explored basic correlations between sensorimotor variables. We reasoned that weathervaning must rely on the detection of temporal changes in the odor concentration. Figure 7 reports how the instantaneous reorientation rate is related to the sensory experience measured at the head (*1/C dC/dt*). In line with the results of Figures 2C,D, 7A indicates that larvae are capable of biasing their runs toward the odor gradient even when they undergo negative sensory experience (black line). This correlation is inexistent when larvae are tested in an arena devoid of odor (light gray line). As lateral head casts have been shown to direct turns in the larva (Gomez-Marin et al., 2011), we examined their potential contribution to the control of weathervaning affecting runs. A visual inspection of the head and tail trajectories reveals that larvae engaged in a run carry out low-amplitude head casts independently of the rest of the body (Supplementary Figure 7). In Figure 7B, we report how changes in odor concentration measured during runs correlate with the head angle. Contrary to the expectation that all head angles directed toward the local gradient (positive values) lead to positive changes in concentration, we find that small head angles toward the gradient and head aligned postures are associated with a negative sensory experience. This observation prompted us to carefully inspect the relationships between the head angle dynamics, the sensory experience and the instantaneous reorientation rate (weathervaning correction) on a representative run illustrated in Figures 8A,B.

In Figure 8, we display the time course of the sensory experience at the head (*1/C dC/dt*, Figure 8A) and the local curvature (ϕ, Figure 8B) of a typical run. Light-gray windows mark segments of the run subjected to stronger reorientation (weathervaning, timestamps 1–4), whereas the dark-gray window indicates the wide-amplitude turn (event denoted by a star). The time courses of the sensory input and the behavioral output highlight that the curvature rate of the run undergoes phases of stronger correction that are preceded by a positive change in odor concentration. By closely examining the positions occupied by the head during the run, we find frequent deviations of the head trajectory (colored line, Figure 8B) with respect to the tail (black line, Figure 8B). During these low-amplitude head casts, the larva undergoes a negative sensory experience when the head points toward the side of lower concentrations. Upon the detection of negative changes in odor concentration, the larva's head tends to swing toward the opposite side—the direction of the higher concentrations (Figures 8B,C). This contralateral cast induces a positive sensory experience (Figure 8D), which reinforces the weathervaning correction toward the side of the local gradient (Figure 8E). Although this model will remain speculative until proven by artificially manipulating the olfaction inputs of the larva by means of optogenetics (Kocabas et al., 2012), the orchestration of the sequence of events underlying weathervaning suggests that active sensing is central to the control of this complex process.

What is the typical signal detectable during low-amplitude and high-amplitude head casts? To characterize the physical scales on which larval chemotaxis operates, we estimate the typical concentration difference measured during the odor-tracking process. Let us consider the situation where the larva is oriented perpendicularly to the gradient at the position where the gradient is maximum. From the sensory landscape reconstruction shown in Supplementary Figure 8, we observe that the slope of the gradient is approximately 0.13 μ M/mm at a point where the concentration is 1.6 μ M. As illustrated in Supplementary Figure 8D, a high-amplitude head cast typically produces a relative body bend of 60°. As the length of a third instar larva can be approximated to 4 mm and the body segment is assumed to bend about half the length of the animal, such a high-amplitude cast would lead to a lateral deflection of the head of 2 mm perpendicularly to the body axis. This spatial difference corresponds to an estimated concentration difference ΔC of 260 nM between the head-bent and head-aligned positions. During head casting to one side, the OSNs located at the tip of the head would experience a relative difference in concentration ΔC/C of about 16%. Now, given a low-amplitude head cast associated with a small body bend of 20° (Figure 8C), we apply the same logic to conclude that the corresponding ΔC is 90 nM, which yields a relative concentration difference of 6%. Therefore, a 1-s head sweep would imply a relative sensory contrast of 0.05/s, which is consistent with the order of magnitude in Figure 8D.

Given that the left and right dorsal organs are separated by less than 150 μm (Cobb, 1999), the inter-nasal concentration difference yields a concentration difference of 20 nM. The relative change in concentration between the left and right OSNs would decrease to approximately 1%. From behavioral and calcium imaging measures (Asahina et al., 2009), we know that the sensory threshold of wild type larvae to ethyl butyrate is on the order of 1 nM, which is lower than the left-right concentration difference. In contrast, head casts generate concentration differences ten-fold larger than those measured between the left and right olfactory organs. In line with previous work, these theoretical estimates and the use of fly genetics allow us to conclude that bilateral sensing is not necessary to direct weathervaning and turns. We propose that low-amplitude head casts during runs and high-amplitude head casts preceding turns are the leading mechanisms contributing to the detection of relative changes in stimulus intensity during chemotaxis (Louis et al., 2008; Gomez-Marin et al., 2010).

In humans, insects, and other animals, orientation behavior is controlled by the sensorimotor transformation of sensory inputs collected through motion (Webb, 2004). For optomotor behaviors, a mechanism akin to efference copy enables flies to tell apart the detection of changes in the stimulus due to the environment from the changes induced by the fly's motion (Heisenberg and Wolf, 1988; Wolf and Heisenberg, 1990). There is a scope to study the neural implementation of this control mechanism during the response of the *Drosophila* larva to spatial odor gradients (Gomez-Marin and Louis, 2012). Curved runs and turns are oriented based on the stimulus dynamics detected during head casts of low and high amplitudes, respectively. A positive change in odor concentrations leads to the acceptance of the direction probed by the head (Gomez-Marin et al., 2011; Gershow et al., 2012). This model appears to be valid for the orientation strategy of the larva to other sensory modalities, including vision and thermosensation (Luo et al., 2010; Kane et al., 2013).

**Figure 9:**
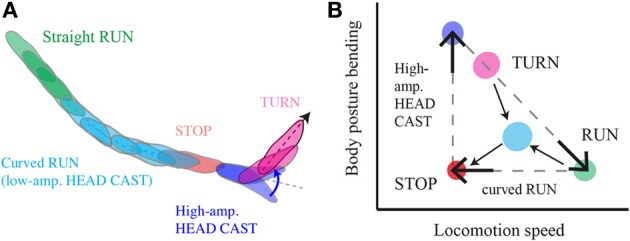
**Multilevel control of progression and reorientation in larval chemotaxis. (A)** Schematic diagram of a typical motor sequence composed of a curved run followed by a turn. Low-amplitude head casts coupled with forward locomotion induce curved runs through weathervaning, interrupted by a stop and large head casts prior to the implementation of a turn. **(B)** Phase-space representation of translational (forward progression) and rotational (body bending) variables that encompasses a kinetic description of larval behavior; the run-stop-cast-turn model approximate a discrete action sequence emerging from a continuum that includes straight runs (green), low-amplitude casts (cyan) and weathervaning (cyan), high-amplitude casts (blue), turns (magenta), and pauses in locomotion (orange). The odor gradient modulates the interplay between behavioral variables in a multilevel manner that constraints the dynamics that real trajectories occupy in the phase-space.

Larval chemotaxis features a series of sensorimotor processes that act in concert (Figure 9). To unravel the mechanisms controlling the orientation of larvae in odor gradients, it is necessary to identify the elementary forms of action underlying this behavior before attempting to relate these actions to plausible sensory-detection and action-selection processes. Such a detailed analysis was conducted for wide-amplitude turns and it considerably improved our understanding of the basic control of turning maneuvers (Gomez-Marin et al., 2011; Gershow et al., 2012). On the other hand, the control of a run involves a combination of two processes. First, the detection of positive temporal gradients suppresses turning whereas the detection of negative gradients facilitates it. Second, the detection of concentration increases through low-amplitude head casts bends the direction of the run. Although both processes involve temporal differentiation of the stimulus, they occur on a different timescale. A head cast can last less than 500 ms, whereas changes in concentration are typically integrated for 5 s before triggering a turn (Figure 4B). These orders of magnitude constrain the dynamic range of the OSN response. Considering the reorientation abilities of larvae with unilateral function in the *Or42b*-expressing OSN (Figure 3), a single OSN should be capable of detecting changes in concentration on a subsecond timescale. The integration of these rapid changes in concentration might be achieved by the olfactory circuitry downstream from the OSNs. In the future, it will be interesting to study how such apparently complex sensory dynamics are represented in the activity pattern of single OSNs. In *C. elegans*, the control of weathervaning and the onset of pirouettes rely on the activity of a sensory neuron that displays an OFF-response (Chalasani et al., 2007; Iino and Yoshida, 2009). Numerical simulations indicate that weathvervaning could emerge from the phasic sensory gating of the left and right motor neurons (Izquierdo and Lockery, 2010). The relevance of these results to the function of the larval OSNs and its coupling to the motor systems remains to be determined.

On the whole, our findings expand the behavioral space that the larva exhibits during chemotaxis. We demonstrate the contribution of smooth corrections in the direction of runs as an integrated component of the hierarchy of sensorimotor processes that control navigation in odor gradients. Rapid progress in our understanding of the organization of locomotion in the larva (Lahiri et al., 2011; Heckscher et al., 2012) and the reconstruction of neural circuits at the level of single synapses (Cardona et al., 2010) make it now possible to identify and functionally characterize the neurons that are part of the sensorimotor pathway underlying larval chemotaxis. The advent of high-throughput behavioral screens will further aid this work (Ohyama et al., 2013). The development of calcium imaging in freely moving animals and closed-loop virtual sensory realities based on optogenetics should permit us to test the sufficiency of the current framework and further establish causal links between sensory, neural and behavioral dynamics (Kocabas et al., 2012; Larsch et al., 2013). In the future, controlling the activity of larval OSNs through optogenetics should allow us to dissect the influence of the parallel and the lateral stimulus gradients on the directional bias and duration of individual runs. Our hypothesis that weathervaning is partly directed by temporal sampling mediated lateral head casts could be tested by manipulating the sensory experience associated with low-amplitude casts occurring during runs. More generally, the study of larval chemotaxis offers a case to decipher how dynamic odorant stimuli are encoded by a single sensory neuron and how this information is transformed downstream to control decision-making and shape actions on the go.

## Author contributions

Alex Gomez-Marin and Matthieu Louis conceived the research and wrote the manuscript. Matthieu Louis generated the UAS-*Or42b* transgene in the Vosshall lab. Alex Gomez-Marin conducted the behavioral experiments and analyzed the data.

## Conflict of interest statement

The authors declare that the research was conducted in the absence of any commercial or financial relationships that could be construed as a potential conflict of interest.
