# Supplementary material for: Multilevel control of run orientation in Drosophila larval chemotaxis
Source: Front Behav Neurosci. 2014 Feb 13;8:38. doi: 10.3389/fnbeh.2014.00038 (PMC3923145; doi:10.3389/fnbeh.2014.00038)
Supplement: Supplementary file 1 [file Presentation1.PDF]

Frontiers in Behavioral Neuroscience

Supplementary material

Multilevel control of run orientation in *Drosophila* larval chemotaxis

Alex Gomez-Marin and Matthieu Louis

Original Research, Front. Behav. Neurosci., 2014, 8:38

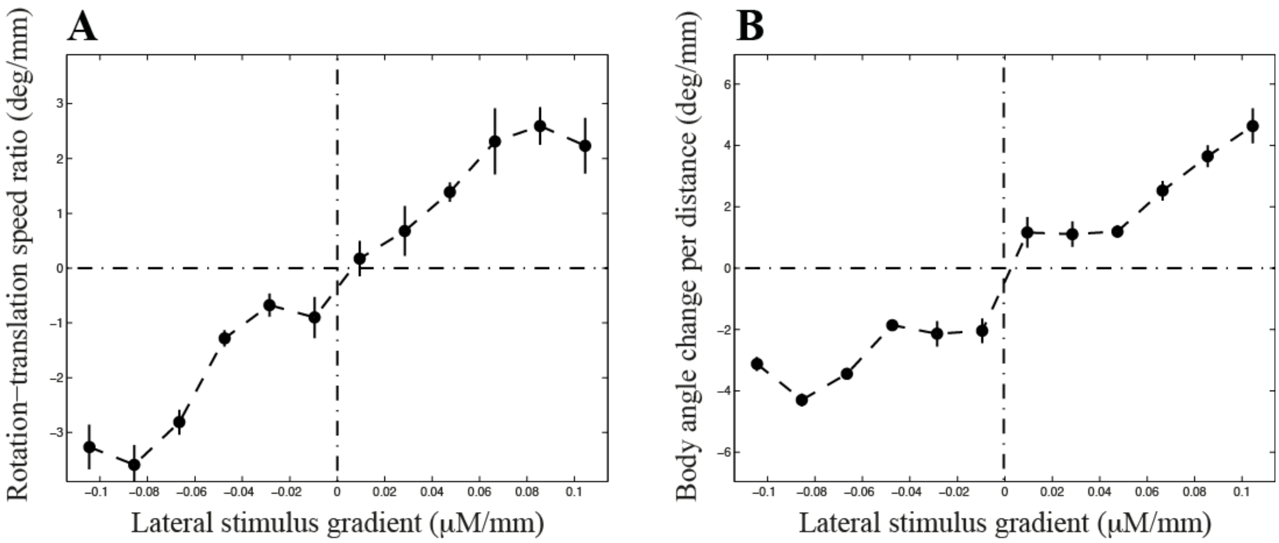

Supplementary Figure 1: **Alternative ways to quantify sensory modulation of curved runs.** The effect of the lateral odor gradient on the local curvature of runs can be quantified through the difference in absolute body angle orientation per unit of time (deg/s) — a kinetic metric called *instantaneous reorientation rate*. Alternatively, differences of body angle per unit of path distance (deg/mm) can be used to account for a more geometrical estimate of curving during runs. The plots depict the effect of the lateral odor gradient on the run curvature when calculated as the instantaneous reorientation rate divided by tail speed **(A)** or, directly, as the change in body angle divided by distance **(B)**. Both metrics have units of deg/mm. They yield the same trend as Figure 2Bi, thus showing that our main results for the weathervane effect do not depend on the definition of the local curvature. Even though we chose to use a kinetic description of curvature throughout the study, geometric descriptions such as those introduced in this figure also capture the weathervane phenomenon.

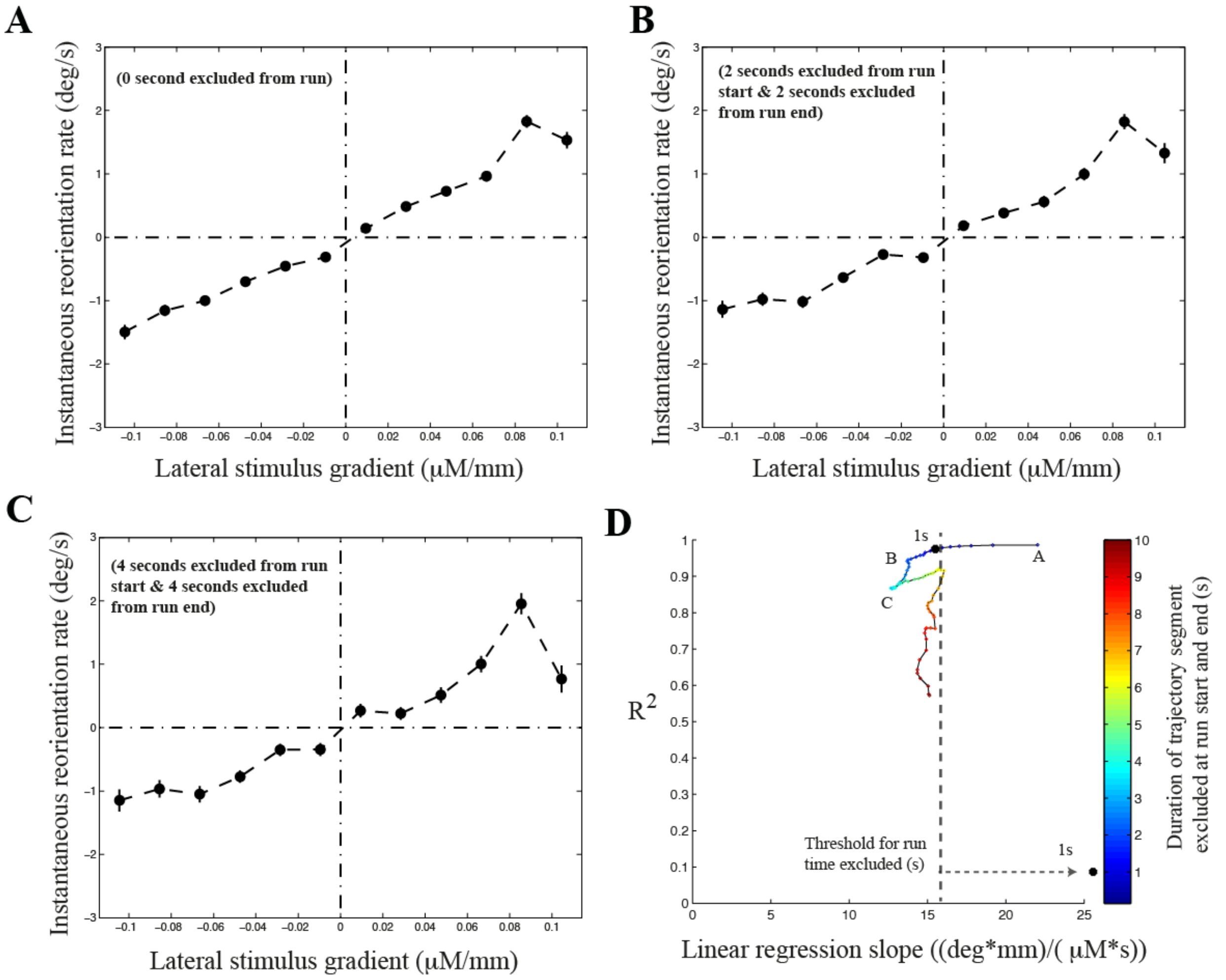

Supplementary Figure 2: Size effect of the exclusion window applied to runs for the quantification of weathervaning.

Mean instantaneous reorientation rate as a function of the lateral odor gradient upon removal of trajectory segments of a fixed duration flanking each run: (A), (B) and (C) are calculated after excluding a time window of 0s, 2s and 4s, respectively, both at the beginning and the end of each run. Main Figure 2Bi corresponds to an exclusion window of 1s, which is the criterion applied throughout the paper unless stated otherwise. Quantifying the strength of the weathervane corrections as a function of the size of the exclusion window permit us to confirm that weathervaning is present in the middle of runs, thereby ruling out artifacts due to the preparation or execution for turning maneuvers. Weathervaning is clearly observed even when only long runs are taken into account (i.e. runs longer than 8 seconds in panel D). **(D)** Quantitative estimation of the optimal threshold on the exclusion window applied to individual runs. Our choice of removing 1s of the trajectory for every run start and run end is justified by considering the linear slope and R^2 coefficient of the linear fit between the reorientation rate and lateral gradient calculated for every duration of run exclusion between 0 and 10s. As shown in the plot, from 0 to 1s the slope of the linear regression quickly decays. Then, it stabilizes as longer run segments are excluded while the correlation becomes weaker. A 1s-window choice represents a trade-off between specificity and strength of the correlation.

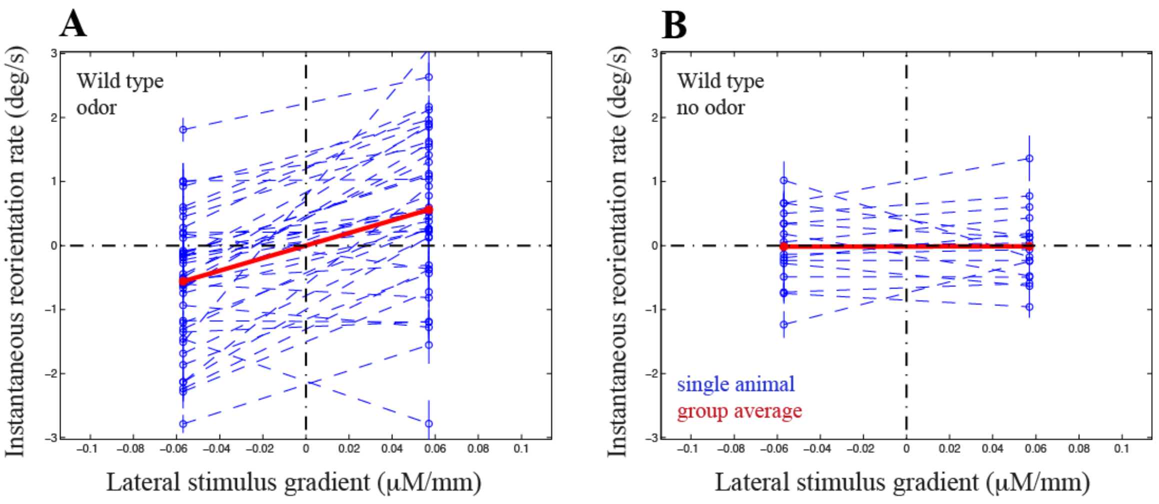

Supplementary Figure 3: Weathervaning effect for individual larvae and averages across animals.
Mean instantaneous reorientation rate for every larva plotted for positive and negative lateral gradients (2 bins). Dashed lines connect the same individual. Error bars represent SEM. Red dots correspond to the average across animals (number of animals used in each condition indicated in the main text). Panels **(A)** and **(B)** represent data for wild type larvae in the presence and absence of odor, respectively. In spite of significant variability across individuals, we find the same trend for positive and negative lateral gradients upon averaging across larvae or pooling all runs. Panel (A) demonstrates the existence of weathervaning in individual larvae stimulated by the odor, while panel B controls for its absence in arenas devoid of odor.

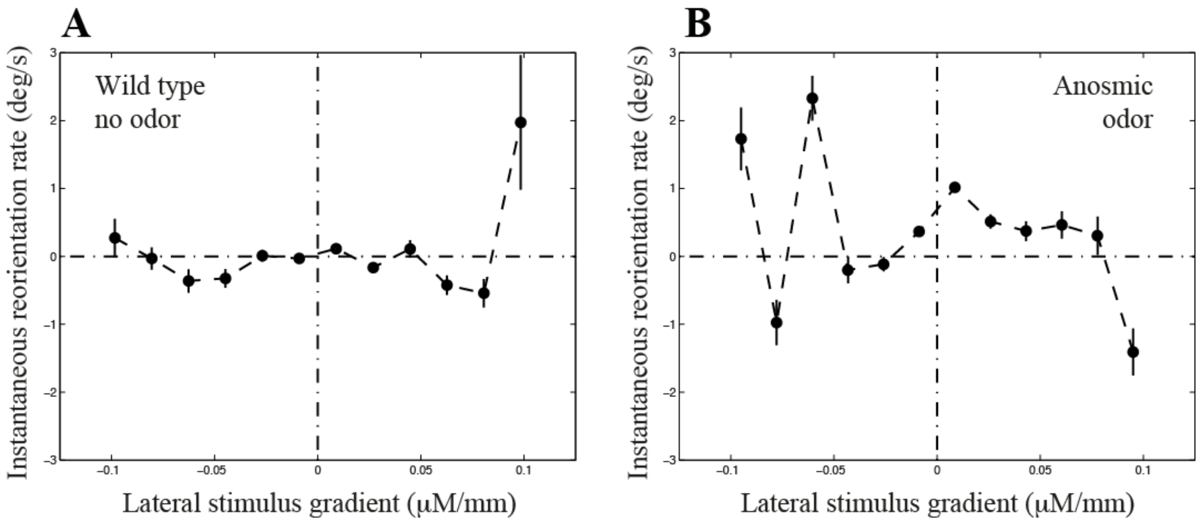

Supplementary Figure 4: Absence of sensory modulation in a silenced olfactory system. Mean reorientation rate versus local sensory gradient shows no significant correlation bias for **(A)** wild type larvae in absence of odor (linear correlation: slope=3.0, R^2=0.089) and **(B)** larvae with an anosmic *Orco* null background in the presence of odor (slope=-6.2, R^2=0.14). These graphs should be compared to the performances of wild type larvae tested in the presence of odor (main Figure 2).

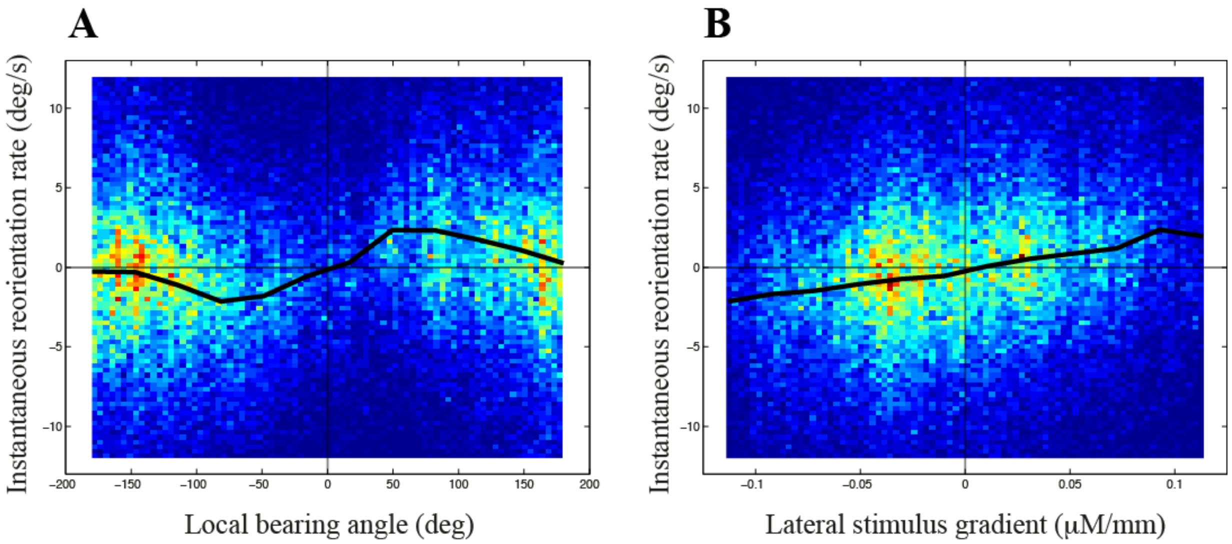

Supplementary Figure 5: Variability and stereotypy in sensory modulation of run directionality.
Instantaneous reorientation rate plotted versus local bearing angle **(A)** and lateral stimulus gradient **(B)**. Solid black lines depict the mean value of the reorientation for the range of sensory parameters clustered in 12 bins of 30 degrees, showing the mean trend of the weathervane correction. Two-dimensional histograms based on the relative occurrence of the data are shown to illustrate the stochastic nature of the modulation. Color bar denotes max (red) and min (blue) probabilities. Wild type larvae (N=42) tested in the presence of odor were used in the analyses of panels (A) and (B). Weathervaning is thus a robust phenomenon that can be quantified through the average reorientation rate.

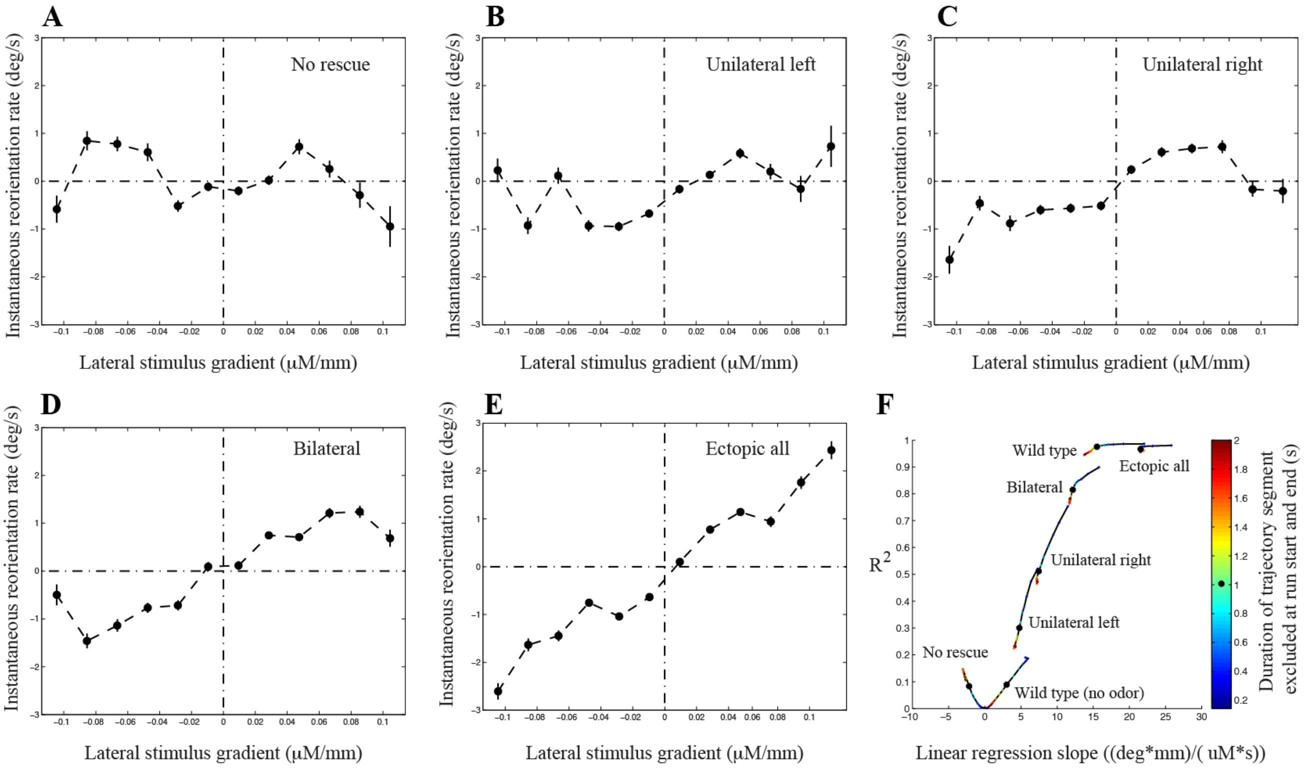

Supplementary Figure 6: Mean reorientation rates as a function of the lateral stimulus gradient for larvae with genetically modified sensory inputs.

Graphs of the mean reorientation rate as a function of the lateral gradient for larvae with modified olfactory systems: no-rescue **(A)**, unilateral left *Or42b* rescue **(B)**, unilateral right *Or42b* rescue **(C)**, bilateral *Or42b* rescue **(D)**, and ectopic expression of *Or42b* in all olfactory sensory neurons **(E)**. Data is clustered in 12 bins of 30 degrees by pooling all animal trajectories during runs (number of animals used in each condition indicated in main text). Error bars represent SEM. While the weathervaning phenomenon is evident for certain groups by looking at the averaged reorientation rates, its existence and strength can be better quantified through a comparison of probability distributions (see main Figure 3). **(F)** Slope and R^2 coefficient of linear correlation computed between the instantaneous reorientation rate and the lateral stimulus gradient. The calculation is repeated as trajectory segments of different lengths are excluded from the beginning and the end of each run. The black dot indicates the exclusion window adopted in the analysis (see Supplementary figure 2 and Methods). A comparison across genotypes demonstrates the dependency of the strength of the weathervane corrections as the number of active olfactory sensory neurons increases.

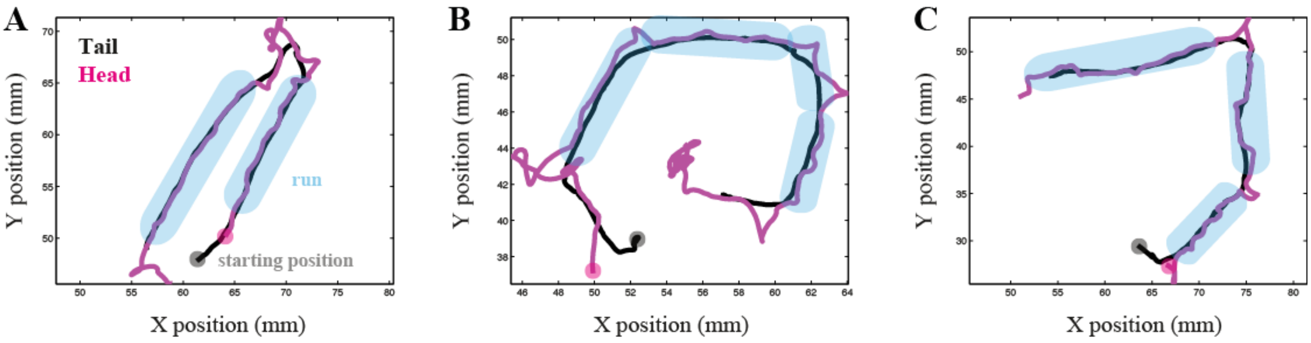

Supplementary Figure 7: Low-amplitude head casts during forward locomotion. Run segments (70s duration) for three different larvae **(A-C)**. While the tail position is relatively smooth (black), the head (magenta) describes low-amplitude lateral sweeps during runs (blue background). These illustrative trajectories show that low-amplitude head casts and curved runs can be uncoupled from forward locomotion, which allows the larva to sample the sensory environment through head casts prior to the implementation of turns during pauses and weathervaning during runs (see main Figure 9).

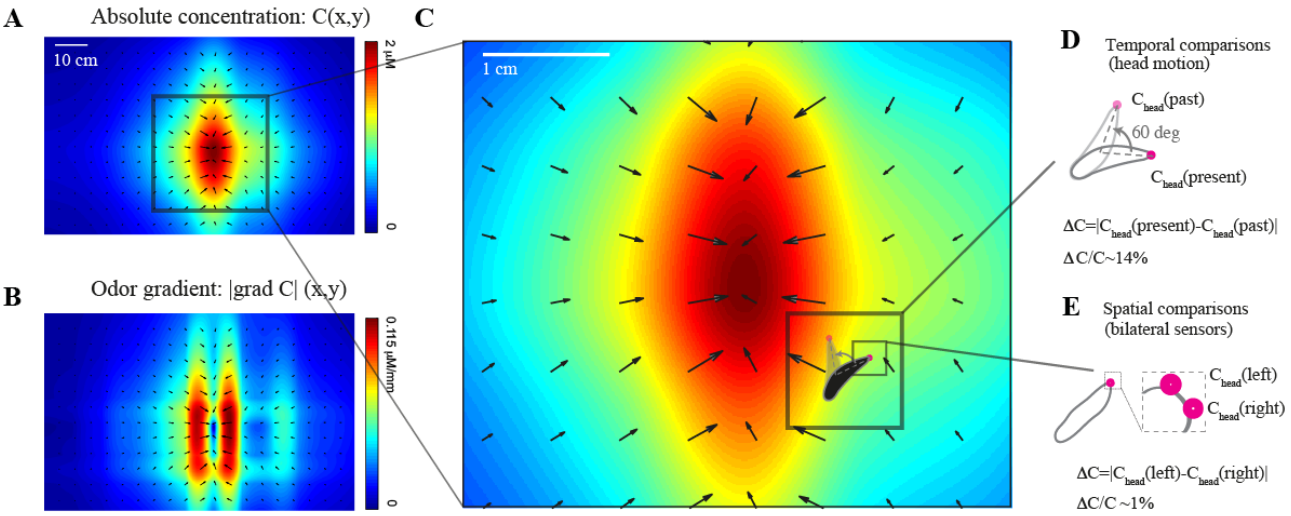

Supplementary Figure 8: Reconstruction of odor gradient and local gradient estimates. **(A)** Spatial map of the absolute odor concentration in the arena. Arrows depict the direction of the gradient. The gradient reconstruction is based on the infrared measurement reported in (Gomez-Marin et al., 2011). **(B)** Spatial map of the strength of the local odor gradient. Arrows depict the direction of the gradient. **(C)** Close-up view of the odor landscape reconstructed at the center of the arena under the odor droplet and overlaid with the body of a *Drosophila* larva that samples the local gradient through lateral head casts. Typical concentration ranges and gradient values that are accessible to the animal can be used to estimate the contribution of spatial sensing (simultaneous bilateral comparisons between sensory organs) and temporal sensing (active sampling through head casts) during larval chemotaxis. **(D)** Order-of-magnitude estimates of the difference in sensory signal acquired at both extremities of a head cast by means of temporal sampling. Head casts account for relative changes in sensory input around 10%. **(E)** Order-of-magnitude estimate of differences in sensory signal associated with instantaneous spatial comparisons between the left and right sensory organs. Bilateral detection of concentration differences in the gradient yields a contrast of 1%.
